# Nanoscale crack initiation and propagation in carbon fiber/epoxy composites using synchrotron: 3D image data

**DOI:** 10.1016/j.dib.2020.105894

**Published:** 2020-06-18

**Authors:** Toshiki Watanabe, Yasuo Takeichi, Yasuhiro Niwa, Masaki Hojo, Masao Kimura

**Affiliations:** a Photon Factory, Institute of Materials Structure Science, High Energy Accelerator Research Organization (KEK), 1-1 Oho, Tsukuba, Ibaraki 305-0801, Japan; b Department of Materials Structure Science, School of High Energy Accelerator Science, SOKENDAI (Graduate University for Advanced Studies), 1-1 Oho, Tsukuba, Ibaraki 305-0801, Japan; c Department of Mechanical Engineering and Science, Kyoto University, Nishikyo-ku, Kyoto 615-8540, Japan

**Keywords:** CFRP, Plastic, X-CT, crack, 3D, Imaging, Synchrotron radiation

## Abstract

Crack initiation and propagation in carbon fiber-reinforced plastic (CFRP) was observed *in situ* under the application of an opening load using nanoscopic synchrotron radiation X-ray computed tomography (nanoscopic SR X-CT) at a high spatial resolution of ∼50 nm. Two datasets of reconstructed and segmented images were produced in typical regions, namely in the thin and thick epoxy regions where the resin thickness between the adjacent carbon fibers was small and large, respectively. This novel study presents the first non-destructive three-dimensional (3D) visualization of resin deformation behavior around crack tips, and provides a valuable and unique insight for the future design of CFRPs.

**Specifications Table**

**Table:** 

**Subject**	Ceramics and Composites
**Specific subject area**	Nano-mechanics of carbon fiber-reinforced plastic (CFRP)
**Type of data**	Image
**How data were acquired**	The data were obtained by X-ray computed tomography (X-CT) using synchrotron radiation with phase-contrast imaging [1]. The CT measurements were conducted at the NW2A beamline of the Photon Factory Advanced Ring (PF-AR) synchrotron facility using a nanoscopic synchrotron radiation (SR) X-CT instrument (Institute of Materials Structure Science, High Energy Accelerator Research Organization (KEK), Japan) [2].
**Data format**	Raw: reconstructed 3D data; tiff Analyzed: segmented 3D data; tiff and avi
**Parameters for data collection**	X-ray energy = 8.0 keV (monochromatic); Voxel size = 39 nm; FOV = 20 μm diameter, 20 × 2 μm length; Number of radiographs = 674; Range of rotation angle = 0–150°; Exposure time = 5 s; Total scan time = *∼*1 h.
**Description of data collection**	X-CT measurements were performed by rotating a nanomechanical test stage with a high-precision piezo actuator (load capacity = 5 N) [3]. A radiograph image with a 20 × 20 µm^2^ was measured by 4 × 4-pixel binning, resulting in a voxel size of 39 nm. Rotation was limited to 150° because the X-ray beam was partially blocked by the bar supporting the wedge indenter.
	
**Data source location**	Photon Factory, Institute of Materials Structure Science, High Energy Accelerator Research Organization (KEK) 1–1 Oho, Tsukuba, Ibaraki 305–0801 Japan
**Data accessibility**	Kimura, Masao; Watanabe, Toshiki; Takeichi, Yasuo; Niwa, Yasuhiro; Hojo, Masaki (2020), “Nanoscale crack initiation and propagation in carbon fiber/epoxy composites using synchrotron: 3D image data”, Mendeley Data, V1, doi: 10.17632/wxmt9tdgh4.1
**Related research article**	T. Watanabe, Y. Takeichi, Y. Niwa, M. Hojo, M. Kimura, Nanoscale *in situ* observations of crack initiation and propagation in carbon fiber/epoxy composites using synchrotron radiation X-ray computed tomography, Compos. Sci. Technol. **197** (2020) 108244.

## Value of the data

This study presents the first non-destructive three-dimensional (3D) evaluation of resin deformation behavior around crack tips. Nano-scale observations were achieved, where spatial resolution has previously been limited to the micro-scale (μm) using other conventional techniques.

The acquired data provides valuable and fundamental insight regarding the related materials (*e.g.* carbon fiber-reinforced plastic (CFRP) and other composite materials and plastic) and theoretical aspects (*e.g.* mechanics and micro-mechanics, especially fracture and fatigue) for further research and engineering applications).

The design of future CFRPs can be based on this data, which provides valuable information on crack initiation and propagation behaviors at the nanoscale. Until now, only theoretical studies or simpler approximation models have been available.

The data present two major mechanisms, namely fiber/plastic interface debonding and in-resin crack initiation. Both mechanisms were found to play crucial roles, and must be considered in order to control crack initiation and to determine reasonable safety margins for the use of CFRP composites.

## Data description

### Raw data

(a) Thin_raw.tif and (b) Thick_raw.tif

Reconstructed 3D images of typical regions, namely the thin and thick epoxy regions in which the resin thickness between the adjacent carbon fibers is small and large, respectively.

Segmented data:

(a-1) Thin_seg_fiber.tif and (a-2) Thin_seg_crack.tif

(b-1) Thick_seg_fiber.tif and (a-2) Thick_seg_crack.tif

The reconstructed data were segmented into carbon fibers, cracks, and resin. Each dataset includes segmented images of carbon fibers and cracks in the thin and thick epoxy regions. The remaining voxels correspond to resin. Segmentation was performed automatically only. In the full paper [1], further manual segmentation was performed to further refine the shapes of the carbon fibers.

Movie data:

(A) MovieS1_CFRP_thin.avi and (B) MovieS2_CFRP_thick.avi

MovieS1_CFRP_thin.avi and MovieS2_CFRP_thick.avi present the 3D automatically segmented data which is composed of carbon fibers (black), resins (dark yellow), and cracks (red) for “thin” and “thick” epoxy regions, respectively. The size of FOV is 20 μm in diameter and 40 μm in length.

## Experimental design, materials and methods

Carbon fiber/epoxy prepreg samples were used with a fiber areal weight of 190 g/m^2^ and resin content of 35 wt.% at a fiber volume fraction of *ca.* 60%. A polyacrylonitrile (PAN)-based carbon fiber with an elastic modulus of 294 GPa and tensile strength of 5880 MPa (T800S, Toray Industries, Inc., Japan) was used as the reinforcing fiber, while a 453 K cure-type epoxy resin compound was used as the matrix resin. Very thin unidirectional laminates [0_2_] were autoclave-cured in a standard cure cycle at a ramp rate of 1.5 K/min and held for 2 h at 450 K and 6 atm. The autoclave-cured CFRP was cut along the fiber direction (*i.e.* fibers were aligned in the Z-direction) using a razor blade to produce columnar samples (length = 1 mm; diameter = 60-μm diameter) for the nanoscopic SR X-CT measurements.

**Fig. 1:**
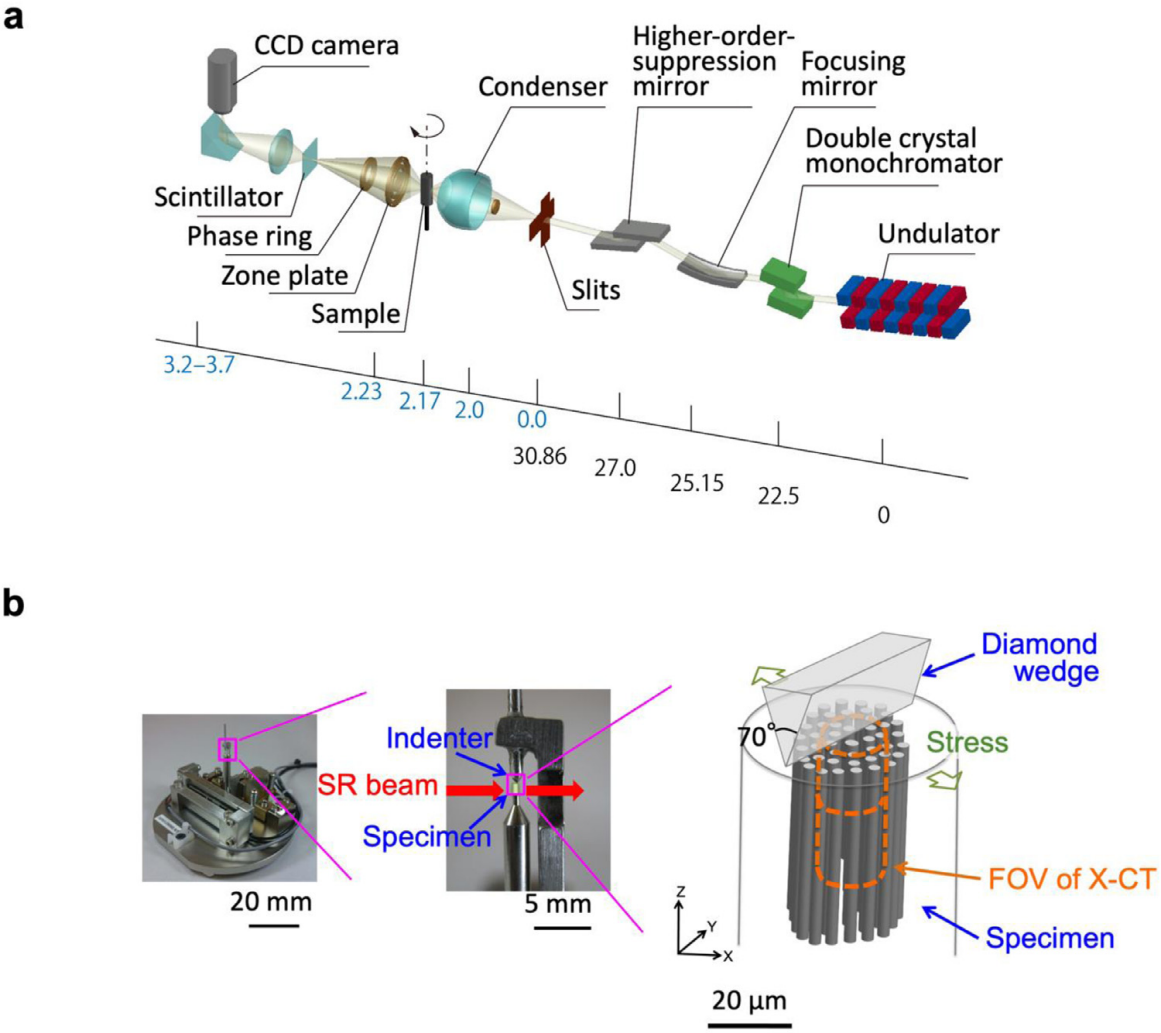
Experimental setup [2]. **(a)** Diagram of the SR X-CT instrument, where black and blue numbers are guides for scales in meter (note: scale is not linear). **(b)** Digital photographs (left) and schematic illustrations (right) of the nanomechanical test stage. The FOV of the nanoscopic SR X-CT measurements (orange lines) was much smaller than the sample dimensions (grey lines), thus eliminating surface effects in the acquired image.

The nanoscopic SR X-CT setup (Fig. 1 [2]) included a monochromatic X-ray beam focused onto the sample using an elliptical glass capillary. The image was projected onto the scintillator using a Fresnel zone plate (ZP) lens with a magnification of ∼70× at 8 keV and was further magnified by an optical lens (∼20×). The image was recorded using a charge-coupled device (CCD) camera, where a total spatial resolution of ∼50 nm was determined by the Siemens star pattern. Phase-contrast imaging relies on the scattering of X-rays at interfaces to enhance image contrast at the fiber/plastic interface, and was thus performed using a phase ring located between the ZP and the scintillator.

The radiographs were reconstructed to 3D volume images using a filtered back projection method. The 3D volume images were segmented, where the Watershed Segmentation feature of the Morphological Segmentation plug-in the Fiji software [4] was used for the cracks, and a deep learning approach in SegNet [5] and MATLAB was used for the carbon fibers.

## Ethics statement

The authors have read and understood the journal's ethical requirements and we believe that neither the article nor the study violates any of these.

## Declaration of Competing Interest

The authors declare that they have no known competing financial interests or personal relationships which have, or could be perceived to have, influenced the work reported in this article.
